# Household Crowding and Neurocognitive Performance in a Cohort of Middle‐Aged Latina Women: The CHAMACOS Maternal Cognition Study

**DOI:** 10.1002/alz.093206

**Published:** 2025-01-09

**Authors:** Jacqueline M Torres, Lucia Calderon, Katherine Kogut, Maria Teresa Rodriguez, Monica Romero, Lizari Garcia, Yesli Perez Rocha, Nadia Rojas, Liliana Paloma Rojas‐Saunero, Brenda Eskenazi, Hector M González

**Affiliations:** ^1^ University of California San Francisco, San Francisco, CA USA; ^2^ University of California, San Francisco, San Francisco, CA USA; ^3^ University of California, Berkeley, Berkeley, CA USA; ^4^ La Clínica de Salud del Valle de Salinas, Salinas, CA USA; ^5^ UC Berkeley School of Public Health, Berkeley, CA USA; ^6^ UCLA, California, CA USA; ^7^ University of California, San Diego, La Jolla, CA USA

## Abstract

**Background:**

Household crowding is a longstanding focus of public health research; there is growing interest in its impact on dementia risk. In the US, household crowding is prevalent among low‐income immigrant communities that have historically been excluded from dementia research. We evaluated the relationship between household crowding and neurocognitive performance among a novel cohort of middle‐aged and primarily immigrant Latinas living in an underserved agricultural community and whether mental health or sleep duration might serve as potential mechanisms.

**Method:**

We used data from the 2022‐2023 CHAMACOS Maternal Cognition Study (n = 513). Household crowding (yes/no) was measured as with binary indicators of > 1 person per habitable room (ppr) and > 2 people per bedroom (ppb); we also considered continuous versions of these measures and the number of household residents < 18 years. Global and domain‐specific cognitive performance z‐scores were measured using the SOL‐INCA cognitive performance battery. Respondents also completed the 10‐item Centers for Epidemiologic Studies ‐ Depression and the 7‐item Generalized Anxiety Disorder scales. Self‐reported hours of nightly sleep were calculated from typical sleep and wake times. We estimated linear regression models controlling for socio‐demographic characteristics.

**Result:**

Participants had a mean age of 48.8 years (SD: 5.2); 22.4%‐20.9% experienced household crowding depending on the metric (> 1 ppr; > 2 ppb). Household crowding (> 1 ppr) was associated with poorer memory (Coef: ‐0.20, 95% CI: ‐0.39, ‐0.02), executive function (Coef: ‐0.21, 95% CI: ‐0.32, ‐0.09), verbal fluency (Coef: ‐0.18, 95% CI: ‐0.33, 0.01) and global cognitive performance (Coef: ‐0.19, 95% CI: ‐0.31, ‐0.07) z‐scores. Associations were consistent when using alternative household crowding measures. Household crowding was also associated with fewer hours of sleep (Coef: ‐0.45, 95% CI: ‐0.80, ‐0.11), although associations with depressive and anxiety symptoms were null.

**Conclusion:**

We observed meaningful associations between household crowding and neurocognitive performance scores among middle‐aged Latina women, with sleep duration as a potential underlying mechanism. Household crowding may be a predictor of future dementia risk and dementia inequities. Reduction of household crowding may be an amenable to population and community‐level intervention (e.g. housing and economic policies) to improve adult cognitive functioning.

